# Histoplasmosis of the Central Nervous System: A Case Series between 1990 and 2019 in French Guiana

**DOI:** 10.4269/ajtmh.20-1486

**Published:** 2021-05-10

**Authors:** Loïc Epelboin, Aïda Dione, Alexandra Serris, Denis Blanchet, Bastien Bidaud, Gaëlle Walter, Philippe Abboud, Emilie Mosnier, Mélanie Gaillet, Céline Michaud, Pierre Couppié, Magalie Demar, Mathieu Nacher, Félix Djossou, Antoine Adenis

**Affiliations:** 1 Infectious Diseases Department, Centre Hospitalier de Cayenne, Cayenne, French Guiana;; 2 CIC INSERM 1424, Centre Hospitalier de Cayenne, Cayenne, French Guiana;; 3 Ecosystèmes Amazoniens et Pathologie Tropicale, EA3593, Université de Guyane, Cayenne, French Guiana;; 4 Centre Gratuit D’Information de Dépistage et de Diagnostic, Centre Hospitalier Agen Nerac - Hôpital Saint-Esprit, Agen, France;; 5 Université Paris Descartes, Centre D’Infectiologie Necker Pasteur, IHU Imagine, Hôpital Necker-Enfants Malades, Assistance Publique-Hôpitaux de Paris (AP-HP), Paris, France;; 6 Laboratory of Parasitology and Mycology, University of French Guiana, Cayenne, French Guiana;; 7 Health Centres for Remote Areas, Andrée Rosemon Hospital, Cayenne, French Guiana;; 8 Dermatology Department, Andrée Rosemon Hospital, Cayenne, French Guiana

## Abstract

Disseminated histoplasmosis is the most frequent acquired immunodeficiency syndrome–defining illness in French Guiana. Paradoxically, central nervous system (CNS) involvement has been scarcely described. We aimed to identify CNS histoplasmosis in our territory. We conducted an observational, multicentric, descriptive, and retrospective study including patients with proven or probable CNS histoplasmosis according to the European Organization for Research and Treatment of Cancer/Invasive Fungal Infections Cooperative Group and the National Institute of Allergy and Infectious Diseases Mycoses Study Group (EORTC/MGS). The study population consisted of patients admitted in one of the hospitals of French Guiana between January 1, 1990 and December 31, 2019. During the study period, 390 cases of HIV-associated histoplasmosis were recorded, in which six of them had CNS infections with *Histoplasma capsulatum*. The male to female sex ratio was 0.25, and the median age at diagnosis was 37.5 years. The median CD4 count was 42 cells/mm^3^ ([IQR: 29–60]). All patients had disseminated histoplasmosis. Usual signs of meningitis were observed in three patients and focal signs in four patients. One patient had no neurological signs. The median time between the first cerebral symptoms and diagnosis was 22.4 days (IQR 9.5–36.2). Two patients died within a month after diagnosis. In conclusion, few proven CNS localizations of histoplasmosis were observed on 30-year study in French Guiana. This low proportion suggests that the documentation of CNS involvement is often not ascertained for lack of awareness of this particular presentation, and for lack of rapid and sensitive diagnostic tools.

## INTRODUCTION

Progressive disseminated histoplasmosis has been an AIDS-defining infection since 1987.^1^ In Latin America, it is considered as one of the most common yet often undiagnosed opportunistic infection of persons with advanced HIV.^2^ As the level of immunity declines, the fungal dissemination worsens, which leads to polymorphous nonspecific manifestations that often involve the lungs, bone marrow, liver, lymph nodes, digestive system, skin, mucosa, genitourinary tract, adrenal glands, bones, joints, etc. Central nervous system (CNS) infection is rarely described. In the 1950s, autopsy studies proved the dissemination of histoplasmosis infection to the CNS. In that study, only two of six patients had had signs of meningitis. Its prevalence has, however, been estimated to concern 5–10% of the patients with disseminated histoplasmosis.^3^ Symptoms are classically subacute over several weeks, with only a quarter of patients having had symptoms for less than a month. The most common manifestations are headaches (60%), altered mental status (42%), focal neurological deficits (30%), cognitive deficits, seizures, and chronic meningitis (39%).^4^

In 60% of cases, the diagnosis of chronic *Histoplasma* meningitis is often delayed by over 1 month, mostly because physicians overlooked the diagnosis. However, more recently, in the United States, the use of *Histoplasma* antigen detection or anti-*Histoplasma* antibody detection in the cerebrospinal fluid (CSF) has led to earlier diagnoses. Whereas the relapse rate and mortality of CNS histoplasmosis used to be very high^5^—often presenting as meningitis in severely immunocompromised patients with poor compliance—antifungal therapy has transformed this universally fatal illness to a manageable condition, if diagnosed and treated early.^6^

*H. capsulatum* is endemic in French Guiana.^7,8^ In this region, disseminated histoplasmosis is the most frequent AIDS-defining illness.^7^ Although physicians are prompt to think about the diagnosis in most cases, and direct examination and culture are available, up-to-date antibody and antigen detection tests are still not routinely available and practiced in the CSF of people living with HIV in French Guiana. Few data regarding CNS histoplasmosis in persons living with HIV are available in the Amazon region. Our objective was thus to document all the cases of CNS histoplasmosis that occurred in our hospital during the last 30 years.

## METHODS

### Study type.

The study was observational, multicentric, descriptive, and retrospective.

### Study site.

The study involved the hospital wards caring for HIV-infected patients requiring hospitalization in the three hospitals of our French Territory of America (Cayenne, Kourou, and Saint Laurent du Maroni).

### Study population.

The source population consisted of people living with HIV hospitalized for an episode of histoplasmosis between January 1, 1990 and December 31, 2019. Patients were identified through the retrospective cohort of the French Hospital Database on HIV. The target population consisted of patients having presented with *H. capsulatum* infection of the CNS during their hospital stay.

Cases were classified as proven or probable CNS histoplasmosis according to the EORTC/MGS criteria (European Organization for Research and Treatment of Cancer/Invasive Fungal Infections Cooperative Group and the National Institute of Allergy and Infectious Diseases Mycoses Study Group). The criteria for proven histoplasmosis were histopathology or direct microscopy on specimens obtained from an affected site showing the distinctive form of the fungus, recovery by culture of the fungus, or blood culture that yields the fungus; criteria for probable invasive histoplasmosis were evidence for geographical or occupational exposure (including remote) to the fungus and compatible clinical illness and *Histoplasma* antigen in urine, serum, or body fluid.^9^ A patient with a positive PCR in the CSF was considered as a putative case. We collected clinical, biological, and radiological data from medical records.

All adults (> 18 years of age) living with HIV with a CNS sample positive for *H. capsulatum* on direct examination and/or culture and/or PCR were included.

### Data collection.

CNS histoplasmosis cases were identified from the database of the laboratory that identified positive CSF samples by mycological or PCR examination. The database of the medical information department of the three hospitals was also queried. For included patients, data were retrospectively collected in the three hospitals of French Guiana from patients’ medical records. Data collection included sociodemographic variables (gender, age, and country of birth), medical data (including general condition, fever, pulmonary signs, digestive signs, and lymphadenopathies), imagery data (brain computed tomography [CT] scan and magnetic resonance imaging [MRI]), routine blood count and CSF results, microbiological data (direct examination, culture, pathology, and PCR), immuno-virological data (CD4 and CD8 counts, and HIV viral load before antiretroviral therapy and at the time of *H. capsulatum* infection), therapeutic data (antifungals, antibiotics, corticosteroids, antiretrovirals...), and the last consultation date after the considered *H. capsulatum* infection.

### Statistical analysis.

The data from the study were analyzed using Microsoft Excel 2013. Descriptive analysis allowed describing sociodemographic, clinical, immuno-virological, and therapeutic data. Means and SDs, or medians and interquartile ranges were used depending on variable distribution.

### Ethical and regulatory aspects.

All HIV-infected patients followed at Cayenne Hospital are included in the DAT'AIDS database. DAT'AIDS (www.dataids.org) is a nonprofit organization created in 2006 around a Scientific Council aiming to compile and analyze databases on HIV, HBV, HCV, STI, and associated pathologies. Data are routinely entered by physicians and research technicians through the eNADIS^®^ computerized medical records. These medical records are used for the management of patients suffering from a chronic pathology, mainly HIV and viral hepatitis. This database is part of the French Hospital Database on HIV, a national retrospective cohort which was described elsewhere.^10^ This database received approval from the Commission Nationale Informatique et Libertés since November 27, 1991. All included patients signed an informed consent form.

## RESULTS

During the 30-year study period, 390 cases of HIV-associated histoplasmosis were recorded in our database. Among them, we identified six CNS infections with *H. capsulatum* (1.5% [95% CI: 0.5–3.3]). Five cases were confirmed cases, and one was probable. Between 2011 and 2019, only one patient with CNS histoplasmosis was identified. The clinical characteristics of the patients are summarized in Table 1. All patients came from endemic areas (two from Haiti, one from Brazil, one from French Guiana, one from Guyana, and one from Suriname). Four were women (sex ratio M:F 0.25), and the median age at diagnosis was 38 years (IQR: 35–45) (Table 2). All had a CD4 count of less than 150 cells/mm^3^ (median CD4 count: 42 cells/mm^3^ [IQR: 29–61]). It was their first episode of histoplasmosis, and no patient was receiving histoplasmosis primary prophylaxis using itraconazole. Diagnosis of HIV infection was already known for all the patients, and three of them were treated: one had been treated by highly active antiretroviral therapy (HAART) for 6 months apparently with good compliance, but the other two had stopped their treatment (Table 3).

**Table 1:** Summary of the cases

Case	1	2	3	4	5	6
Age (years) gender	33 Female	36 Female	47 Male	35 Male	48 Female	39 Female
Country of birth	Haiti	Haiti	Guyana	Suriname	French Guiana	Brazil
Year of the onset of symptoms	1993	1998	2007	2008	2009	2014
Delay between diagnoses of HIV and cerebral histoplasmosis (month)	52	57	0	18	48	6
Duration between 1st symptoms and diagnosis (days)	17	98	0	7	28	39
CD4 count (cells/mm^3^)	29	62	29	7	65	56
HIV RNA (copies/mL)	UK	UK	UK	687,000	59,894	147,615
HAART at presentation	No	No	No	Yes but nonobservant: ritonavir, fosamprenavir, abacavir, and lamivudine	Yes but nonobservant: zidovudine, lamivudine, lopinavir, and ritonavir	Tenofovir, lamivudine, and efavirenz
Coinfection	HCV	HCV	None	None	None	None
Opportunistic diseases	No	No	Cerebral toxoplasmosis and pulmonary TB	Oral candidiasis	No	CMV viremia and pulmonary TB
Non-CNS symptoms (duration)	Fever, diarrhea, and short breath (12 weeks)	Fever and splenomegaly (2 weeks)	Weight lost, cough, and diffuse lymphadenopathy (12 weeks)	Weight lost, fever, abdominal pain, vomiting, and bronchial rattel (12 weeks)	Weight lost, dyspnea, cough, expectoration, and otomastoiditis	Fever, abdominal pain, nausea, vomiting, diarrhea, and cough (20 weeks)
CNS symptoms (duration)	Meningoencephalitis	Meningeal syndrome, and headaches (2 weeks)	Headaches, ideomotor slowdown, and focal deficit	No	Ataxia	Headaches, confusion, temporospatial disorientation, meningeal syndrome, and dysarthria (2 weeks)
Imaging	Chest X-RAY = interstitial syndrome	Chest X-RAY = right upper lobe opacity sequelae of tuberculosis	Brain CT scan = edema and parenchymatous lesions in the cockade	Brain CT scan = normal	Brain CT scan = normal	Brain CT scan = hypodense gaps of the left internal capsule
	Abdominal US = normal	Ab echo = splenomegaly and lymphadenopathy				
	Brain CT scan = not performed	Brain CT scan = hydrocephalus				MRI = hypersignals of the region of the central gray nuclei in injected T1 and FLAIR
CSF analysis	UK	WBC: 71	WBC: 20 (% P: 5, L: 95)	WBC: 1	WBC: 44 (%P: 25, L: 75)	WBC: 11
		PR: 0.98 g/L	PR: 1,4 g/L	PR: 0.3 g/L	PR: 1.7 g/L	PR: 0.72 g/L
		GLU: 2.2 mmoL/L	GLU: 2.9 mmoL/L	GLU: 2.8 mmol/L	GLU: unknown	GLU: 1.2 mM
CSF culture	Positive	Positive	Negative	Positive	Positive	Positive
Other positive culture to *H. capsulatum*	Bone marrow Blood	Bone marrow	Bone marrow	Bone marrow	Bone marrow	Bone marrow Blood
PCR on CSF	Not performed	Not performed	Positive	Negative	Negative	Not performed
Anti-TB treatment initiated before histoplasmosis diagnosis	Yes	Yes	No	No	No	Yes
Treatment	IV AmB	Oral itraconazole	IV L-AmB for 7 days and then oral itraconazole	Itraconazole	IV L-AmB	IV AmB for 1 day and then oral itraconazole
Modified HAART regimen	Zidovudine	No	No	Ritonavir, fosamprenavir, lamivudine, and abacavir	Didanosine, zidovudine, ritonavir, and lopinavir	Ténofovir, emtricitabine, and efavirenz
28-Day death	Yes	No	No	No	Yes	No
Outcome (month)	1	88	96	53	0	78

CMV = cytomegalovirus; CSH = cerebrospinal fluid; IV = intravenous; GLU = glycorrhachia; HAART = highly active antiretroviral therapy; L = lymphocytes; L-AmB = liposomal amphotericin B; MRI = magnetic resonance imaging; P = polynuclear neutrophils; PR = proteinorrachia, TB = tuberculosis; UK = unknown; WBC = white blood cells.

**Table 2:** Summary of the main continuous variables of the six patients and comparison to the French Guiana histoplasmosis cohort

Continuous variable	Central nervous system histoplasmosis, 1990–2019 (*N* = 6)			Disseminated histoplasmosis, 1990–2019 (*N* = 279)			
	Median	IQR: 25–75	Range	Median	IQR: 25–75	Range	*N*
Age (years)	38	35–45	33–48	39	34–46	19–68	279
Duration between first symptoms and diagnosis (days)	23	10–36	1–69	NA	NA	NA	NA
Viral load	147,615	103.755–417,308	59,894–687,000	385,000	98,487–623715	66–8200,000	52
CD4	42	29–61	7–65	32	12–72	0–550	274
Delay between diagnoses of HIV and cerebral histoplasmosis (month)	33	9–51	0–57	24	0–72	0–252	220

**Table 3:** Summary of the main categorical variable of the six patients and comparison to the French Guiana histoplasmosis cohort

Categorical variables	Central nervous system histoplasmosis, 1990–2019 (*N* = 6), *n* (%)	Disseminated histoplasmosis, 1990–2019 (*N* = 279), *n* (%)
Male gender	2 (33)	184 (66.0)
Highly active antiretroviral therapy at the diagnosis	3 (50)	28 (10.0)
28-Day death	2 (33)	35 (12.5)

All patients had disseminated histoplasmosis, with a positive bone marrow culture for 5/5 of them and positive blood cultures for two of them.

Two of them had a coinfection with hepatitis C virus, and four of them had another opportunistic infection (tuberculosis, the Epstein–Barr virus, Cytomegalovirus, and cerebral toxoplasmosis).

Usual signs of meningitis including headache, nuchal rigidity, and fever were observed in three of six patients. A focal sign (such as motor deficit or ataxia) was observed in four patients. One patient had no neurological signs. The median time between the first cerebral symptoms and diagnosis was 23 days (IQR: 10–36). Five of six CSF culture were positive. The remaining case was diagnosed by PCR in the CSF. Bone marrow was positive in mycological culture for all patients and blood culture for two patients. Biochemical characteristics of the CSF were available for five patients. Four of five had biological meningitis with lymphocytic predominance, hyperproteinorrachia, and hypoglycorrhachia. The median hemoglobin level was 89 g/L (86–104), and the median neutrophil count was 1.7 G/L (1.6–1.9). Beta-D-glucan was only performed in patient #6 supported in 2014, and the level was 412 pg/mL (normal < 80). Galactomannan antigen detection was performed in none of them.

Five patients had a cerebral imaging (among the five patients with CT scan, one also had an MRI): two patients presented a hydrocephaly, and one had parenchymal lesions compatible with fungal abscesses. Two patients had a normal cerebral scan. MRI was performed in one patient with hypersignals of the region of the basal ganglia in injected T1 and FLAIR. Antituberculosis treatment was started for three of the six patients before obtaining the mycological diagnosis.

Three patients were treated with liposomal amphotericin B (L-Amb), one with deoxycholate amphotericin B, and two with itraconazole as a first-line treatment. One patient was switched from L-Amb to itraconazole after 1 week because of poor tolerance. The duration of treatment is not known. No surgical intervention was performed.

Zidovudine was introduced for the patient treated in 1993, which did not prevent his death. Highly active antiretroviral therapy was modified for patients 4, 5, and 6, but this did not prevent the death of the first two patients, which was not due to an immune reconstitution inflammatory syndrome (IRIS). Patient 6 did not develop IRIS and is still alive 5 years after her management, with immuno-virological success, but a persistent neurological symptomatology—such as headache—and abnormal CSF with a proteinorachy between 1 and 1.5 g/L and a cellulorachy around 30 cells per mm^3^, predominantly lymphocytic, without any other infectious cause was found, despite extensive explorations.

Two patients died within a month after diagnosis, and two others died from a bacterial infection 4 and 7 years after diagnosis, respectively. One patient remained with persistent headaches and CSF abnormalities for several years despite persistent immune restoration.^11^ Two patients were still alive at the time this manuscript was written.

## DISCUSSION

We report six cases of CNS histoplasmosis in HIV-infected patients in French Guiana. This represents 1.5% of disseminated cases over the study period, which is far lower than what was reported in the United States. The most likely explanation for our observation is that, despite the fact that physicians are quite aware of the major importance of *H. capsulatum* among opportunistic infections, diagnosis is still difficult in the absence of valuable diagnostic methods such as antigen detection, serodiagnosis, or PCR in the CSF.^12^ Neurological signs are nonspecific and may not be on the forefront of the clinical presentation and may even be absent. Cerebrospinal fluid analysis can be normal in highly immunocompromised patients. CNS histoplasmosis should be considered as a serious differential diagnosis of CNS tuberculosis, which is one of the main causes of CNS involvement in AIDS. One of our patients had no clinical signs of meningitis, a normal CSF white blood cell count, and a normal cerebral CT scan. Fungal culture from CSF has poor sensitivity, and it is slow, thus delaying the onset of treatment. PCR is a useful tool, allowing earlier diagnosis in this life-threatening disease, but it is not always performed. Diagnostic delay is an important obstacle for optimal treatment of CNS histoplasmosis. A complementary explanation of the low prevalence may be that when the diagnosis of disseminated is performed from samples from another organ (bone marrow, biopsies…), treatment is often already presumptively given, or is promptly started, and the exhaustive list of the dissemination sites may seem to be relatively trivial compared with the diagnosis itself. However, this is not true for CNS involvement because of the therapeutic repercussions of this specific site infection. Recommendations suggest liposomal AmB 5 mg/kg/day should be given for up to 4–6 weeks as tolerated, followed by oral itraconazole for at least 12 months. Because of the possibility of relapses, CSF parameters should be reevaluated before discontinuation of itraconazole at 12 months to verify that cell counts are normal and, when possible, that *Histoplasma* antigen is absent from the CSF. When the CNS involvement is not known, transition from L-Amb to itraconazole, and then itraconazole interruption may be accelerated, thus leading to suboptimal treatment of the CNS infection.^6^

Although it has been suggested that there are genetic and phenotypic differences between *H. capsulatum* clades in the United States and South America, we do not believe our observation reflects virulence differences.

In conclusion, we observed few proven CNS localizations in French Guiana. This low proportion suggests that the documentation of CNS involvement is often not ascertained for lack of awareness of this particular presentation, and for lack of rapid and sensitive diagnostic tools. This leads to suboptimal treatment strategies that treat disseminated histoplasmosis without taking into account the requirements of the neurological dissemination. Hence, although clinicians are very aware of the importance of disseminated histoplasmosis in French Guiana, there is still a margin of progress, which reemphasizes the need for antigen detection tests in all countries where *H. capsulatum* is endemic.^13^ In front of a clinical picture of subacute or chronic encephalitis in a person living with HIV, one should evocate the diagnosis of histoplasmosis of the CNS, as a possible differential diagnosis of tuberculosis of the CNS.
